# Improving Adherence and Clinical Outcomes in Self-Guided Internet Treatment for Anxiety and Depression: A 12-Month Follow-Up of a Randomised Controlled Trial

**DOI:** 10.1371/journal.pone.0089591

**Published:** 2014-02-25

**Authors:** Nickolai Titov, Blake F. Dear, Luke Johnston, Peter M. McEvoy, Bethany Wootton, Matthew D. Terides, Milena Gandy, Vincent Fogliati, Rony Kayrouz, Ronald M. Rapee

**Affiliations:** 1 Centre for Emotional Health, Department of Psychology, Macquarie University, Sydney, Australia; 2 Centre for Clinical Interventions and School of Psychology, UWA, Perth, Australia; Federal University of Rio de Janeiro, Brazil

## Abstract

**Background:**

A recent paper reported the outcomes of a study examining a new self-guided internet-delivered treatment, the *Wellbeing Course*, for symptoms of anxiety or depression. This study found the intervention resulted in significant symptom reductions. It also found that automated emails increased treatment completion and clinical improvements in a subsample with elevated anxiety and depression.

**Aims:**

To examine the clinical outcomes and the effect of automated emails at 12 months post-treatment.

**Method:**

Participants, who were randomly allocated to a Treatment Plus Automated Emails Group (TEG; n = 100), a standard Treatment Group (TG; n = 106) or delayed-treatment Waitlist Control Group (Control; n = 51), were followed up at 12 months post-treatment. Eighty-one percent, 78% and 87% of participants in the TEG, TG and treated Waitlist Control Group provided symptom data at 12-month follow-up, respectively. The primary outcome measures were the Patient Health Questionnaire-9 Item Scale (PHQ-9) and the Generalized Anxiety Disorder-7 Item Scale (GAD-7).

**Results:**

Significant improvements in symptoms of anxiety and depression were observed over time in both the TEG and TG (*F*s >69, *p*s <.001) these were sustained from post-treatment to 12-month follow-up (*p*s >.05), and were associated with large effect sizes. No statistically significant differences in symptoms were found between the TEG and TG at post-treatment, 3-month or 12-month follow-up. Previously reported symptom differences between TEG and TG participants with comorbid symptoms were no longer present at 12-month follow-up (*ps* >.70).

**Conclusions:**

The overall benefits of the Wellbeing Course were sustained at 12-month follow-up. Although automated emails facilitated Course completion and reductions in symptoms for participants with comorbid anxiety and depression from pre-post treatment, these differences were no longer observed at 12-month follow-up. The results indicate that automated emails promote more rapid treatment response for people with elevated and comorbid symptoms, but may not improve longer term outcomes.

**Trial Registration:**

Australian and New Zealand Clinical Trials Registry ACTRN12610001058066

## Introduction

Self-guided internet-delivered psychological interventions for anxiety and depressive disorders have considerable public health potential. However, more than 90% of consumers typically withdraw after two sessions [1], [2] and, consequently may not receive a dose of therapeutic content sufficient to lead to sustained improvement. Self-guided internet interventions that include strategies to facilitate adherence have considerable potential for optimizing outcomes. Trials of self-guided internet interventions for depression [3], [4] and social phobia [5], [6], [7] show that automated reminder emails increased completion rates and effect sizes relative to earlier trials without automated reminders. Automated emails may be particularly suitable as a medium for providing reminders given their ubiquity and low cost.

A recent paper reported an evaluation of a new self-guided internet-delivered treatment, the *Wellbeing Course*, for symptoms of anxiety or depression [8]. Participants had no contact with clinicians prior to or during treatment and were randomly allocated to receive the Wellbeing Course administered with automated emails (Treatment Plus Automated Email Group: TEG, n = 100), without automated emails (Treatment Group: TG, n = 106) or to a Waitlist Control Group (Control Group; n = 51), who subsequently received treatment. At post-treatment, participants in the two Treatment Groups obtained similar and significant improvements on measures of anxiety and depression relative to the Control Group. However, those participants receiving automated emails obtained higher rates of course completion, defined as having read all five lessons of the course, than those not receiving emails (58% v. 35%). While no overall differences in symptom improvement were found between the Treatment Groups, a subgroup of participants with comorbid anxiety and depression, that is, participants with elevated symptoms of both anxiety and depression, obtained superior reductions in symptoms when they received automated emails compared to when they did not receive the emails. The pattern of these results was sustained at 3-month follow-up.

An outstanding question is whether these results are sustained in the longer term. Return or exacerbation of symptoms may occur following treatment for anxiety disorders and depression [9], [10]. The primary aim of the present study was to test the hypothesis that the benefits observed at post-treatment and 3-month follow-up would be maintained at 12-month follow-up. Previous results indicate that benefits of guided internet interventions for anxiety or depression [11], [12], [13] are sustained in the longer-term, but limited data exist for longer-term benefits of self-guided internet interventions [14]. An additional aim was to examine the number of participants who deteriorated at 12-month follow-up, defined as an increase in PHQ-9 or GAD-7 scores of 5 or more points from pre-treatment. No formal hypothesis was generated with respect to deterioration, but this data was collected to provide information about the safety of the Wellbeing Course.

## Methods

### Ethics Statement and Trial Registration

The original study [8] was approved by the Human Research Ethics Committee (HREC) of the University of New South Wales (UNSW, Sydney, Australia) and ratified by the Macquarie University HREC (Sydney, Australia; HREC Reference: 5201100226). The original protocol included telephone interviews at recruitment and a fourth group who received weekly contact with a therapist. However, in order to focus on the benefits of providing automatic emails during self-guided treatment and to simulate an entirely automated treatment process, the interviews and this fourth group were omitted from the final design. The trial was registered with the Australian and New Zealand Clinical Trials registry as ACTRN12610001058066. The protocol for this trial and supporting CONSORT checklist are available as supporting information; see Checklist S1 and Protocol S1.

### Design and Sample Size

The original trial randomly allocated 257 participants to one of two Treatment Groups or to a Waitlist Control Group. Participants in the Treatment Plus Automated Email Group (TEG; n = 100) received access to the Wellbeing Course with automated emails. Participants in the Treatment Group (TG; n = 106) received access to the Wellbeing Course without automated emails. The Control Group (Controls; n = 51) received access to the same treatment as the TEG following post-treatment; the data presented here are the data for the Control Group in Treatment, that is, after the Two Treatment Groups completed treatment and the Control Group received treatment.

### Measures

The primary outcome measures were depression and anxiety symptom severity, as measured by the PHQ-9 and GAD-7. The GAD-7 has good convergent validity with other anxiety scales [15]–[17], is sensitive to DSM-IV congruent generalised anxiety disorder, social phobia and panic disorder, with increasing scores indicating greater symptom severity [16]. A total score of 8 on the GAD-7 has been identified as an important threshold for identifying the presence of an anxiety disorder [15]–[17]. A total score of 10 on the PHQ-9 has been identified as an important threshold for identifying DSM-IV congruent depression with increasing scores indicating greater symptom severity [15], [17]-[20]. Additional information collected comprised demographic details including: age, gender, educational qualifications, vocational status, treatment satisfaction and completion rates (i.e., percentage in each treatment group who read all five online lessons of the Wellbeing Course). Additional measures included changes in health status and service utilisation, which will be reported separately as part of an evaluation of the cost-effectiveness of the intervention.

TEG and TG participants completed the PHQ-9 and GAD-7 at pre-treatment, prior to each of the five lessons, at post-treatment, 3-month follow-up and 12-month follow-up. Control Group participants completed the PHQ-9 and GAD-7 at pre-treatment and post-treatment and, subsequently, commenced the same intervention as the TEG group with data collected at pre-treatment, prior to each lesson, at post-treatment, 3 month follow-up and 12 month follow-up. Their results after they received and completed the treatment phase are presented here as a partial replication in order to test the reliability of the TEG condition.

### Intervention

The Wellbeing Course is a five lesson transdiagnostic internet intervention based on a pragmatic model of psychotherapeutic change that assumes that symptoms of anxiety and depression are the result of unhelpful habits of thought and action, that is, maladaptive cognitions and behaviours [8], [22]. This model also proposes that interventions that are structured, systematically present increasingly complex material over weeks and months, and are sufficiently engaging to achieve adherence and commitment, will result in reductions in unhelpful habits and an increase in adaptive habits. Further, such interventions are likely to result in large and sustained improvements in symptoms compared to sporadic or unstructured therapy sessions, which may only result in short-term symptom relief. More details about the Wellbeing Course can be found elsewhere [8].

### Procedure

TEG and TG participants received an email at the start of the Course providing guidelines and a recommended timetable of Course-related activities. TEG participants also received at least two additional emails per week during the Course. Emails were triggered when (1) participants read a Lesson, (2) if participants had not read a prescribed Lesson within 7 days of it becoming available, (3) at the start of each week, and (4) at times during the Course when participants were known to experience increased symptoms or to have increased difficulties practicing skills (e.g., during the weeks when graded exposure was introduced). Emails were comprised of three or four sentences, used the participant's first name, and employed a warm and supportive tone. The content informed participants about newly available or unread materials, reinforced progress and skills practice, normalised the challenges of learning new skills, and encouraged realistic expectations of recovery. All participants consented to receive the emails and no emails contained personal or detailed clinical information.

Up to five automated email reminders were sent to all participants to complete post-treatment or follow-up questionnaires. These emails were only sent where participants had not completed the questionnaires. Once participants had completed the questionnaires they were sent a brief template-based feedback email detailing their results. At the 3- and 12-month follow-up time points participants who had not completed the questionnaires after the five automated emails were telephoned once by the researchers. No clinical contact was made between the 3- and 12-month follow-up periods and no clinical content was discussed in these telephone calls. The Course material was made available for the 12-month period following the end of the 8-week treatment.

### Statistical methods

Consistent with the initial report of this trial [8], several separate mixed-models analyses were employed to analyse the data at post-treatment, 3-month and 12-month follow-up. These mixed-models employed an autoregressive covariance structure and maximum likelihood estimation, which provides unbiased estimates in the case of missing data; under the assumption that data is missing at random. These mixed-models specified Group (i.e., TEG and TG) and Time (i.e., pre-treatment, post-treatment, 3-month follow-up and 12-month follow-up) as fixed effects with the Intercept set at pre-treatment and Participants specific as a random effect. Primary interest was the Group x Time interactions and differences within and between the two Treatment Groups at the different time points were examined using pairwise comparisons. Consistent with the original report of this study [8], one set of analyses was conducted using the entire data set (i.e., the *Overall Sample*) and a second set of analyses was restricted to participants with elevated symptoms of both anxiety and depression (i.e., the *Comorbid Sample*), defined by pre-treatment scores above clinical cut-offs on both primary outcome measures (i.e., total GAD-7 ≥8 and total PHQ-9 ≥10). Effect sizes (Cohen's *d*) and 95% confidence intervals were calculated for both within-group and between-group effects based on the pooled standard deviation. All analyses were performed in SPSS version 19.0 (SPSS, Inc., Chicago, IL).

As described in recent dissemination studies [23], pre-treatment, post-treatment and follow-up PHQ-9 and GAD-7 scores were compared with clinical cut-offs to provide an index of clinically significant *remission*. This was defined as the proportion of participants who initially scored at or above the clinical cut-offs and then subsequently below the clinical cut-offs. Finally, the proportion of participants with significant deterioration in PHQ-9 or GAD-7 scores were reported, calculated for each group based on a completer analysis and defined as an increase in PHQ-9 or GAD-7 scores of five or more points from pre-treatment [15]. In these analyses, missing values were handled using the Multiple Imputation [21] procedure where Group as well as GAD-7 and PHQ-9 scores at each time point were used to generate 20 sets of imputed data. These 20 imputed data sets where then averaged to provide pooled frequency counts, chi-square statistics and *p* values. All results are reported also presented for the Waitlist Control Group, after they had received treatment, as a partial replication of the results of the TEG group.

## Results

### Adherence and Attrition

Twelve-month follow-up data were collected from 81%, 78% and 87% of TEG, TG and treated Waitlist Control Group participants, respectively.

Between post-treatment and follow-up participants in both treatment groups continued to log in and read the online materials. At the 12-month follow-up there was no longer a significant difference in the number of TEG (68%) and TG (59%) participants who had read the five lessons, that is, completed the Course, *X^2^* (1)  = 1.63, *p* = .20. Similarly, in the Comorbid Sample, no significant difference was found between the TEG (66%) and TG (59%) participants who had completed the Course at 12-month follow-up, *X^2^* (1)  = 0.67, *p* = .41.

### Primary Outcome Measures

**Figure 1:**
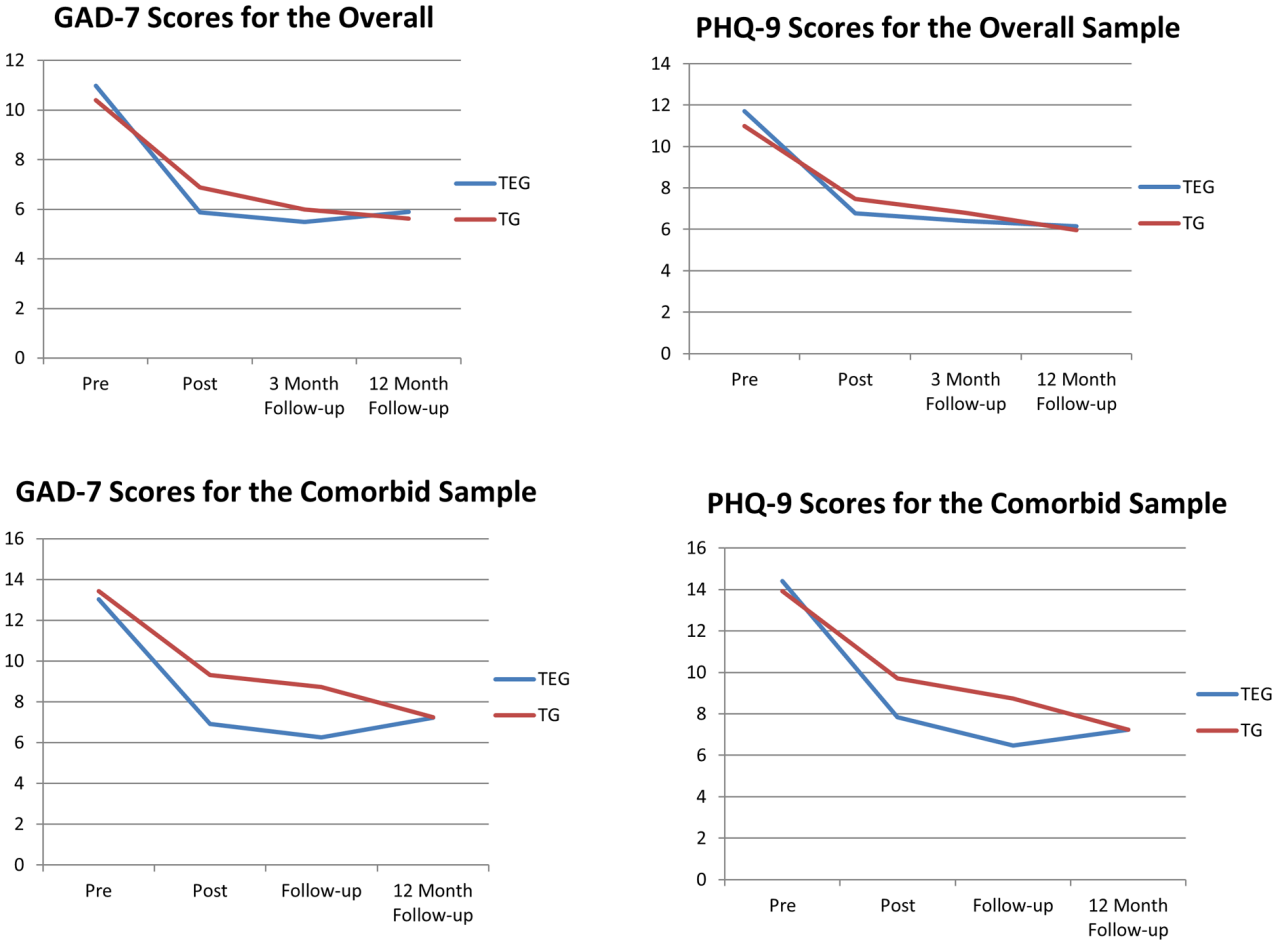
Means for the PHQ-9 and GAD-7 for the Overall and Comorbid Samples. Note: TEG: Treatment plus automated email group. TG: Treatment group. PHQ-9: Patient Health Questionnaire-9 Item. GAD-7: Generalised Anxiety Disorder-7 Item.

Means for the PHQ-9 and GAD-7 at each time point are included in Table 1 and Figure 1 for the Overall Sample. Means for the Comorbid Sample are shown in Table 2 and Figure 1.

**Table 1:** Results of outcome measures for the Overall Sample: Observed and Estimated Means, Standard Deviations, 95% Confidence Intervals and Effect Sizes (Cohen's d) for each group.

	Observed Means				Estimated Marginal Means				Effect sizes (based on estimated marginal means)					
	Pre	Post	3-month Follow-up	12-month Follow-up	Pre	Post	3-month Follow-up	12-month Follow-up	Within Group Pre to Post	Between Post TEG vs. TG	Within Group Pre to 3-month follow-up	Between 3- month follow-up TEG vs. TG	Within Group Pre to 12-month follow-up	Between 12- month follow-up TEG vs. TG
**PHQ-9**														
TEG^a^	11.71 (4.72)	6.73 (4.50)	6.24 (4.42)	6.12 (4.60)	11.71 (4.89)	6.78 (5.27)	6.41 (5.36)	6.14 (5.25)	.97 (0.67–1.26)	0.13 (−0.15–0.40)	1.03 (0.73–1.32)	0.07 (−0.20–0.35)	1.10 (0.80–1.39)	−0.03 (−0.13–0.24)
TG^b^	10.99 (4.92)	7.41 (5.91)	6.38 (5.07)	5.83 (4.49)	10.99 (4.89)	7.46 (5.24)	6.80 (5.34)	5.96 (5.32)	0.70 (0.42–0.97)	–	0.82 (0.53–1.10)	–	0.98 (0.69–1.27)	–
**GAD-7**														
TEG^a^	10.98 (4.23)	5.75 (3.78)	5.51 (4.13)	5.91 (4.18)	10.98 (4.54)	5.87 (4.87)	5.49 (4.94)	5.90 (4.85)	1.09 (0.78–1.38)	0.21 (−0.07–0.48)	1.16 (0.85–1.45)	0.10 (−0.17–0.38)	1.08 (0.78–1.37)	−0.06 (−0.33–0.22)
TG^b^	10.40 (4.98)	6.80 (5.62)	5.60 (4.25)	5.61 (3.98)	10.40 (4.54)	6.88 (4.82)	6.00 (4.91)	5.63 (4.89)	0.75 (0.47–1.03)	–	0.93 (0.64–1.21)	–	1.01 (0.72–1.29)	–
**Waitlist Control Group (In Treatment)** ^c^														
PHQ-9	10.50 (5.04)	5.69 (4.08)	5.67 (4.66)	5.34 (4.67)	10.50 (4.70)	6.08 (4.93)	6.02 (4.89)	6.28 (4.98)	0.92 (0.46–1.36)	–	0.93 (0.48–1.38)	–	0.87 (0.42–1.31)	–
GAD-7	8.76 (4.44)	5.58 (4.17)	4.53 (3.43)	4.75 (3.71)	8.76 (4.23)	5.66 (4.26)	4.67 (4.24)	5.00 (4.37)	0.74 (0.29–1.17)	–	1.07 (0.60–1.51)	–	0.85 (0.40–1.29)	–

*Note.* Standard deviations and confidence intervals are shown in parentheses. Estimated Marginal Means are derived from the mixed-models analyses. TEG: Treatment Plus Automated Email Group. TG: Treatment Group; PHQ-9: Patient Health Questionnaire 9-Item; GAD-7: Generalised Anxiety Disorder 7-Item.

a n = 100 ^b^n = 105 ^c^n = 42

**Table 2:** Results of outcome measures for the Comorbid Sample (cut-offs on both PHQ-9 and GAD-7): Observed and Estimated Means, Standard Deviations, 95% Confidence Intervals and Effect Sizes (Cohen's *d*) for each group.

	Observed Means				Estimated Marginal Means				Effect sizes (based on estimated marginal means)					
	Pre	Post	3-month Follow-up	12-month Follow-up	Pre	Post	3-month Follow-up	12-month Follow-up	Within Group Pre to Post	Between Post TEG vs. TG	Within Group Pre to 3-month follow-up	Between 3- month follow-up TEG vs. TG	Within Group Pre to 12-month follow-up	Between 12- month follow-up TEG vs. TG
**PHQ-9**														
TEG^a^	14.40 (3.27)	7.78 (4.51)	6.39 (4.51)	7.16 (4.89)	14.40 (4.52)	7.84 (4.92)	6.48 (5.03)	7.23 (4.82)	1.39 (0.98–1.78)	0.38 (0.01–0.75)	1.66 (1.23–2.06)	0.44 (0.07–0.81)	1.53 (1.12–1.93)	0.00 (−0.37–0.37)
TG^b^	13.93 (3.30)	9.75 (6.02)	8.41 (5.94)	7.12 (4.34)	13.93 (4.53)	9.72 (4.99)	8.74 (5.20)	7.24 (5.05)	0.98 (0.57–1.37)	–	1.06 (0.01–0.75)	–	1.39 (0.96–1.80)	–
**GAD-7**														
TEG^a^	13.02 (3.63)	6.76 (4.09)	6.30 (4.18)	6.74 (4.46)	13.02 (4.37)	6.93 (4.73)	6.26 (4.82)	7.00 (4.65)	1.34 (0.93–1.72)	0.50 (0.13–0.87)	1.47 (1.06–1.86)	0.42 (0.04–0.78)	1.33 (0.93–1.72)	0.06 (−0.31–0.43)
TG^b^	13.43 (3.80)	9.31 (5.98)	7.92 (4.47)	7.32 (4.49)	13.43 (4.36)	9.31 (4.75)	8.29 (4.95)	7.28 (4.82)	0.90 (0.50–1.29)	–	1.10 (0.69–1.50)	–	1.34 (0.91–1.75)	–

*Note.* Standard deviations and confidence intervals are shown in parentheses. Estimated Marginal Means are derived from the mixed-models analyses. TEG: Treatment Plus Automated Email Group. TG: Treatment Group; PHQ-9: Patient Health Questionnaire 9-Item; GAD-7: Generalised Anxiety Disorder 7-Item.

a n = 60 ^b^n = 54

#### Overall Sample

The mixed-model analyses examining GAD-7 scores for the Overall Sample revealed a significant effect for Time (*F_3, 467_* = 87.57, *p*<.001), but not for Group (*F_1, 212_* = 0.11, *p* = .74) or the Time by Group interaction (*F_3, 467_* = 1.27, *p* = .28). Pairwise comparisons revealed no significant differences between the TEG and TG groups at post-treatment, 3-month follow-up or 12-month follow-up (*p* range  = .14 to .69). Both the TEG and TG groups improved significantly from pre-treatment to post-treatment (*p*<.001), but did not obtain any further significant changes from post-treatment to 3-month follow-up (*p*>.05) or from 3-month follow-up to 12 month follow-up (*p*>.05). However, the scores of both groups remained significantly lower at 3- and 12-month follow-up compared with pre-treatment (*p*<.001), indicating that symptom improvements were maintained.

Similarly, the mixed-model analyses examining PHQ-9 scores for the Overall Sample revealed a significant effect for Time (*F_3, 471_* = 74.48, *p*<.001), but not for Group (*F_1, 219_* = 0.01, *P* = .93) or the Time by Group interaction (*F_3, 471_* = 0.79, *p* = .50). Pairwise comparisons revealed no significant differences between the TEG and TG groups at post-treatment, 3-month follow-up, and 12-month follow-up (*p* range  = .35 to .81). However, both the TEG and TG groups improved significantly from pre-treatment to post-treatment (*p*<.001), but did not obtain any further significant changes from post-treatment to 3-month follow-up (*p*>.05) or from 3-month follow-up to 12-month follow-up (*p*>.05). The scores of both groups were sustained and remained significantly lower at 3- and 12-month follow-up compared with pre-treatment (*p*<.001).

#### Comorbid Sample

The mixed-model analyses for the GAD-7 scores for the Comorbid Sample revealed a significant effect for Time (*F_3, 258_* = 69.03, *p*<.001) and Group (*F_1, 126_* = 4.21, *p* = .042), but not the Time by Group interaction (*F_3, 258_* = 1.63, *p* = .18). Pairwise comparisons revealed that both the TEG and TG groups improved significantly from pre-treatment to post-treatment (*p*<.001), but did not obtain any further significant changes from post-treatment to 3-month follow-up (*p*>.05) or from 3-month follow-up to 12-month follow-up (*p*>.05). The scores of both groups were sustained and remained significantly lower at 3- and 12-month follow-up compared with pre-treatment (*p*>.001). Pairwise comparisons also revealed the TEG group had significantly lower scores than the TG group at post-treatment and 3-month follow-up (*p* range  = .01 to .03), but not at 12-month follow-up (*p* = .75).

The mixed-model analyses of the PHQ-9 scores for the Comorbid Sample revealed a significant effect for Time (*F_3, 268_* = 72.58, *p*<.001), but not for Group (*F_1, 138_ = *2.24, *p* = .14) or the Time by Group interaction (*F_3, 268_* = 2.27, *p* = .08). Pairwise comparisons revealed that both the TEG and TG groups improved significantly from pre-treatment to post-treatment (*p*<.001), but did not obtain any further significant changes from post-treatment to 3-month follow-up (*p*>.05) or from 3-month follow-up to 12-month follow-up (*p*>.05). The scores of both groups were sustained and remained significantly lower at 3- and 12-month follow-up compared with pre-treatment (*p*>.001). Pairwise comparisons also revealed the TEG group had significantly lower scores than the TG group at post-treatment and 3-month follow-up (*p* range  = .02 to .04), but not at 12-month follow-up (*p* = .99).

#### Waitlist Control Group (following treatment)

The mixed-model analyses examining the scores of those in the Waitlist Control Group following treatment revealed significant effects for Time on both the GAD-7 (*F_3, 95.38_* = 12.80, *p*<.001) and PHQ-9 (*F_3, 97.99_* = 15.71, *p*<.001). Pairwise comparisons revealed significant improvements on both the PHQ-9 and GAD-7 from pre-treatment to post-treatment (*p*<.001), but no significant changes from post-treatment to 3-month follow-up (*p*>.05) or from 3-month follow-up to 12-month follow-up (*p*>.05). However, 3-month follow-up and 12-month follow-up scores on the PHQ-9 and GAD-7 remained significantly lower than pre-treatment scores (*p*<.001), indicating that the treatment effects were maintained.

### Effect Sizes

Within- and between-group effect sizes for the outcome measures are included in Tables 1 and 2. For both the Overall and Comorbid Samples large within-group effect sizes (i.e., ≥0.80) were found for the TEG and moderate-to-large within-group (i.e., ≥0.50) effect sizes were found for the TG. Similar large within-group effect sizes were observed in the Control Group following treatment. Small and non-significant between-group effect sizes (i.e., ≤0.30) were found between the two Treatment Groups. Small-to-moderate between-group effect sizes were found between the Comorbid Samples (i.e., 0.30 to 0.50) at each of the time points, except at 12-month follow-up time point, when the effect sizes were small and non-significant (i.e., ≤0.30).

### Clinical Significance

#### Overall Sample

As shown in Table 3, post-treatment remission rates for the PHQ-9 and GAD-7 were sustained at the 12-month follow-up time points for the TEG (64.7% and 63.1%) and TG (68.6% and 60.8%).

**Table 3:** Proportion of participants in the Overall and Comorbid Sample, above and below cut-off scores of clinical significance (remission).

Overall Sample	TEG		TG		Chi Square	Waitlist Control Group (In Treatment)^c^	
	N	%	N	%	TGE vs. TG	N	%
**PHQ-9**							
Pre-treatment score > 10/Total	68/100	68.0	67/106	63.2	*X^2^* (1) = 0.52, *P* = .46	22/42	52.3
Post-treatment score < 10 (Remission)/Total	42/68	61.7	42/67	62.6	*X^2^* (1) = 0.11, *P* = .81	14/22	63.6
3-month follow-up score < 10 (Remission)/Total	51/68	75.0	46/67	68.6	*X^2^* (1) = .82, *P* = .48	15/22	68.1
12-month follow-up score <10 (Remission)/Total	44/68	64.7	46/67	68.6	*X^2^* (1) = 0.36, *P* = .62	14/22	63.6
**GAD-7**							
Pre-treatment score > 8/Total	76/100	76.0	74/106	69.8	*X^2^* (1) = 0.99, *P* = .31	22/42	52.3
Post-treatment score < 8 (Remission)/Total	47/76	61.8	46/74	62.1	*X^2^* (1) = 0.17, *P* = .76	10/22	45.5
3-month follow-up score < 8 (Remission)/Total	51/76	67.1	48/74	64.8	*X^2^* (1) = 0.22, *P* = .72	15/22	68.1
12-month follow-up score <8 (Remission)/Total	48/76	63.1	45/74	60.8	*X^2^* (1) = 0.29, *P* = .66	14/22	63.6
**Comorbid Sample**							
Pre-treatment PHQ-9 score > 10 and GAD-7 score > 8/Total	60/100	60.0	54/106	50.0	*X^2^* (1) = 1.70, *P* = .19	19/42	45.2
Post-treatment PHQ-9 score < 10 and GAD-7 score <8 (Remission)/Total	43/60	71.6	38/54	70.3	*X^2^* (1) = 0.09, *P* = .81	14/19	73.6
3-month follow-up PHQ-9 score < 10 and GAD-7 score <8 (Remission)/Total	51/60	85.0	39/54	72.2	*X^2^* (1) = 2.82, *P* = .16	14/19	73.6
12-month follow-up PHQ-9 score < 10 and GAD-7 score <8 (Remission)/Total	44/60	73.0	40/54	74.0	*X^2^* (1) = 0.23, *P* = .71	14/19	73.6

*Note.* All data within table is pooled data from 20 generated sets of imputed data using the Multiple Imputation procedure.

#### Comorbid Sample

As shown in Table 3, the post-treatment remission rates were also sustained at 12-month follow-up for the Comorbid Samples of the TEG (73%) and TG (74%).

#### Waitlist Control Group (following treatment)

The remission rates for the Control Group and the remission rates for the Comorbid Sample of the Waitlist Control Group following treatment were also sustained at 12-month follow-up.

### Deterioration

#### Overall Sample

At 12-month follow-up, up to 3.3% of TEG and 5.0% of TG participants obtained PHQ-9 or GAD-7 scores five or more points higher compared to pre-treatment. No Waitlist Control Group participants had scores five or more points higher after treatment at 12-month follow-up.

#### Comorbid Sample

At 12-month follow-up, 3.0% of TEG and 1.8% of TG participants in the Comorbid Samples obtained PHQ-9 or GAD-7 scores five or more points higher compared to pre-treatment.

## Discussion

The primary finding from this study was that the self-guided Wellbeing Course resulted in clinically significant reductions in symptoms of anxiety and depression at post-treatment and these reductions were sustained at the 3- and 12-month follow-up time points. These findings are consistent with results from clinician-guided internet interventions, which demonstrate that benefits are sustained in the longer term [11], [12] indicating the robust nature of internet interventions. The magnitude of clinical benefits from this self-guided intervention at 12-month follow-up were larger than those reported at follow-up than other self-guided internet treatments for depression or anxiety [12], [24]. Benefits from the present trial also compare favourably with self-guided internet treatments for depression or anxiety where participants had contact with researchers at pre-treatment [25], [26], with trials of transdiagnostic internet interventions involving clinician guidance [27], [28], [29] and trials of clinician-guided transdiagnostic interventions administered face-to-face [30]. However, while the magnitude of gains reported here are encouraging, caution should be exercised in making comparisons across studies due to differences in samples, inclusion criteria, and methodologies.

An important secondary finding of the present study was that the benefits of automated emails, in increasing completion rates and in reducing symptoms in a subsample with elevated and comorbid symptoms, were no longer found at 12-month follow-up. In the present study, at 12-month follow-up, the TG obtained similar outcomes to the TEG on both the PHQ-9 and GAD-7 and both groups evidenced remission rates above 50% at 12-month follow-up in each group. Rates of deterioration at 12-month follow-up appeared larger in the TG, but the overall event rate was low (<5%).

The absence of benefits from automated emails appears to have been the result of continued improvements in the TG group, of whom an additional 25% completed the Course between the 3- and 12-month follow-up points; compared to 7% of the TEG group. Consequently, at 12-month follow-up, there was no difference between groups in the proportion who had read all five lessons or in the clinical outcomes of either the Overall or Comorbid Samples. The reasons for this increase in completion rates in the TG are unclear. However, it is possible that the automated assessment emails at post-treatment and 3-month follow-up prompted TG participants to return to the Course, although it is also likely that participants found the material in the Course sufficiently helpful in the first instance to justify their return.

These results raise the question of whether automated emails are helpful or necessary. Our findings suggest that automated emails facilitated more rapid symptom improvement with the Comorbid Sample who received automated emails relative to the Comorbid sample who did not receive emails. However, the results indicate that the automated emails were not absolutely *necessary* to achieve comparable outcomes in the longer term, as the TG group obtained similar outcomes as the TEG at the 12-month follow-up. This indicates that the decision about whether or not to include automated emails should be informed by the costs, risks and the benefits of their implementation and the value a service places on the speed of clinical improvements. Nevertheless, it is likely that more rapid reductions in symptoms would be associated with reduced health costs and lifetime burden of disease. Therefore, the inclusion of automated emails is recommended.

These results also extend debate and questions about the mechanisms of change in internet interventions [31]. The magnitude of clinical benefits found in this study indicates that, at least for some patients, direct contact with a therapist is not necessary for clinically significant change to occur during internet treatment. This is not to say that therapeutic factors that facilitate engagement and adherence, such as the credibility of an intervention, reinforcement and unconditional positive regard, are not important. Rather, we contend that these and other factors can and should be integrated into the content, format and presentation of self-guided interventions. We suspect that as improvements are made to the content of internet-delivered psychotherapeutic interventions and in the delivery of that content, an increasing proportion of the population will be able to benefit from them. However, we caution against assuming that self-help materials can be easily developed and delivered. Just as therapists require careful training and supervised practice, internet intervention materials should also be carefully developed and improved through a process of repeated clinical evaluation and quality assurance cycles.

We also contend that these results provide support for pragmatic models of psychotherapeutic change, which emphasise the importance of systematically learning, practicing and adopting core psychological skills. These skills support participants to change unhelpful habits of thought and action, which, in turn, have broad positive impacts on emotion dysregulation. We suspect the use of case-enhanced learning strategies (e.g., detailed case studies) in the course content facilitated engagement and adherence. However, as noted by others, multiple other factors such as providing a clear deadline and expectations of feedback also facilitate adherence and clinical outcomes [26]. It appears likely that systematic examination of other strategies to promote engagement and adherence in self-guided internet interventions, including prompts delivered by mobile technologies, will further improve outcomes in self-guided internet-delivered interventions and will also have utility across a broad range of health conditions [32].

### Limitations

These findings need to be considered in the context of the limitations of the overall study. The long-term effects of the study were not assessed alongside an active control group, although the large magnitude of the outcomes indicates there were strong treatment effects. The generalisability of these results is limited by the nature of the sample, which comprised people seeking internet-delivered treatment for anxiety and depression. However, it is important to note that a comparison of demographic and symptom profiles of participants in internet trials with those identified in epidemiological samples indicates that the results of internet trials may generalise to the wider population [33]. Moreover, while symptom improvements were sustained, the present study relied on self-report measures rather than structured diagnostic interviews, which would have required clinical contact and may have affected outcomes. It is also important to note that the present study employed a specific transdiagnostic treatment intervention, the Wellbeing Course, which was designed and developed for use as either an entirely self-guided or clinician-guided Course. Consequently, it is possible that automated emails may facilitate greater adherence and clinical outcomes in less developed interventions or interventions initially developed to involve clinician guidance but subsequently used in a self-guided format.

## Conclusions

The present results indicate that the benefits of the self-guided Wellbeing Course were sustained at 12-month follow-up. The inclusion of automated emails facilitated adherence, completion and more rapid symptom reduction during the Wellbeing Course in the immediate and short-term for those with comorbid symptoms. However, these emails did not improve outcomes any further in the longer term or for the overall group. We contend that automated emails are helpful, particularly for those with elevated symptoms, and should be considered when developing self-guided and clinician-guided interventions where possible. Overall, the Wellbeing Course was clinically effective and acceptable to consumers. These results are encouraging and indicate the public health potential of well-developed self-guided interventions.

## Supporting Information

Checklist S1 **CONSORT Checklist.**
(DOC)

Protocol S1 **Trial Protocol.**
(PDF)
